# Cosmetics

**Published:** 1875-02

**Authors:** 


COSMETICS.—Science has plainly gone backward in the fabrication of substances for whitening and reddening the face. Chalk and pink saucers appear to have been superseded by some sort of liquid preparations, which destroy the beautiful texture of the skin, and give a deathly appearance to the complexion. The whitewash used imparts a blue tint, and makes its user look ghastly and frightful. We intend to find out the name of the stuff, and give it a good notice as the article most destructive of good looks ever known. We hardly know of whom to inquire, as we do not number among our friends one foolish enough to so disfigure herself. But there are multitudes who daily varnish their faces with these destructive compounds, and thus make themselves the subjects of ridicule and contempt. A man who would marry one of these painted creatures would deserve the fate which could not fail to await him.

Fortunately, all good society places its ban upon this class of artists. It is generally understood that only vicious females or very silly girls practice this abominable art. Of course the merely silly ones are not long in becoming vicious, for the step is a short one.

It is curious to see how these artful and artificial persons run in shoals. In New York, the observing resident can tell you just when the painted phalanx will be out in force. You may walk all over the city, morning after morning, when honest girls are going briskly to their daily work, and not meet a kalsomined creature among the multitude. The wretched women who paint their faces, keep late hours and only venture out in pleasant weather. You will meet them any fine afternoon from Union Square to the Fifth Avenue Hotel. They generally seek to attract the attention of men as silly as themselves. They dress in the absurd height of any fashion which may be affected by the French demi-monde. They caricature respectable women in every imaginable way, and call forth from all decent people the scorn which they take such infinite pains to earn.

---

EARLY EDUCATION.—No tongue can tell the power of early education and association upon the life of an individual. They color the whole after life. They are like the daily food eaten by wild game, so pungent and saporific in its nature that it flavors every fibre of their flesh, and colors every bone in their bodies. It is evident, therefore, that while the school properly makes intellectual work its prominent part, it should supplement in moral influence the work of the home, and when the child is morally starved at home, teachers should be impressed with the double responsibility resting upon them in the education of such a pupil. We need not wonder at the immoral bent of so many children when we visit their homes and see the debasing influences there at work. The thoughtful, helping influence of teachers is beyond all price. Lay the foundation well and the superstructure will not fall.

---

Thousands of foolish women neglect the exercise which gives freshness, and then paint their cheeks, and think they look pretty. They simply make laughing stocks of themselves.

